# Abnormalities in Tooth Formation after Early Bisphosphonate Treatment in Children with Osteogenesis Imperfecta

**DOI:** 10.1007/s00223-021-00835-2

**Published:** 2021-03-20

**Authors:** Barbro Malmgren, Irma Thesleff, Göran Dahllöf, Eva Åström, Georgios Tsilingaridis

**Affiliations:** 1 Department of Dental Medicine, Division of Orthodontics and Pediatric Dentistry, Karolinska Institutet, POB 4046, 141 04 Huddinge, Sweden; 2 Developmental Biology Program, Institute of Biotechnology, University of Helsinki, Helsinki, Finland; 3 Center for Pediatric Oral Health Research, Stockholm, Sweden; 4 TkMidt – Center for Oral Health Services and Research, Mid-Norway, Trondheim, Norway; 5 Department of Women’s and Children’s Health, Karolinska Institutet, Stockholm, Sweden; 6 Pediatric Neurology, Astrid Lindgren Children’s Hospital at Karolinska University Hospital, Stockholm, Sweden

**Keywords:** Bisphosphonates, Tooth abnormalities, Enamel, Tooth morphology, Tooth agenesis, Osteogenesis imperfecta

## Abstract

Treatment with intravenous bisphosphonate (BP) in children and adolescents with osteogenesis imperfecta (OI) started in Sweden in 1991. No human studies on the role of BP therapy in development of disturbances in tooth mineralization or tooth morphology have been published. The study cohort comprised 219 individuals who were divided into four groups: group 1, BP treatment onset before 2 years of age (*n* = 22); group 2, BP treatment onset between 2 and 6 years of age (*n* = 20); group 3, BP treatment onset between 6 and 10 years of age (*n* = 13); and a control group of patients with OI who had not received BP therapy (*n* = 164). The chi-square test was used in between-group comparisons of the prevalence of tooth agenesis. The prevalence of tooth agenesis was significantly higher in children who began BP treatment before the age of 2 years (group 1; 59%,) compared to the controls (10%; *p* < 0.001) and to children who had begun BP therapy between ages 2 and 6 years (group 2; 10%; *p* = 0.009) or between ages 6 and 10 years (group 3; 8%; *p* = 0.003). Different types of disturbances in the enamel formation were seen in 52 premolars, where 51 were seen in those who began BP treatment before the age of 2 years. To conclude, starting BP treatment before the age of 2 years increases the risk of abnormalities in tooth formation manifesting as morphological aberrations, tooth agenesis, and enamel defects.

## Introduction

Osteogenesis imperfecta (OI) is a clinically and genetically heterogeneous group of heritable disorders of connective tissue which is caused by dominant mutations in collagen type I, encoded by *COL1A1* and *COL1A2* in up to 90% of cases [1]. Clinical signs may include bone fragility, short stature, joint laxity, hearing loss, tendency to prolonged bleeding, bruising, blue sclera, and dentinogenesis imperfecta (DGI) [2] as well as other dental abnormalities like tooth agenesis and malocclusions [3, 4]. OI has traditionally been classified in four main types based on primary clinical and radiological findings combined with pattern of inheritance: type I (mild OI with blue sclera), type II (pre- or perinatal lethal), type III (severe deforming), and type IV (intermediate severity and white sclera after infancy) [5]. This classification is now expanded into five different phenotype groupings type 1–5 where type 5 is characterized by calcification of the interosseous membranes and/or hypertrophic callus [6]. The prevalence of OI types I, III, and IV in Sweden has been estimated at 7.4/100,000 [7].

Treatment with intravenous pamidronate (APD) started in Sweden in 1991 in children and adolescents with OI with repeated fractures, vertebral compression, and pain [8]. Treatment was initially given to adolescents with OI type III. Later, younger children were successively included and treatment indications over time changed to include infants with more severe forms of OI (repeated fractures and acquired vertebral compressions) and also children with milder OI with progressive vertebral fractures, low bone density, and pain. Similar treatments have been started elsewhere and treatment with bisphosphonates (BPs) in patients with OI has expanded and has become an important symptomatic therapy especially in moderate and severe OI [9]. The rationale for BP therapy of OI is predominantly inhibitory effect on osteoclasts which results in a net increase in bone mass and mineralization [10]. A substantial body of work has demonstrated the efficacy of these agents in preventing bone fractures, decreasing pain, and increasing the quality of life [11]. Intravenous, i.v., pamidronate, neridronate, or zoledronate is the treatment of choice for pediatric patients with moderate-to-severe OI, whereas BP treatment for patients with mild forms of OI is still discussed [12–14]. In most previous studies, the average age of children with OI at onset of BP therapy is around 4 years, although there are reports of children who started treatment as early as 2 weeks of age [15]. During the first four years of age, the permanent teeth are under intense development and theoretically BP therapy could affect the tooth formation.

Earlier, we reported a high prevalence of tooth agenesis in individuals with OI (17%) [4] and impairment of the maturation and eruption of permanent teeth when pamidronate has been administered from infancy [16]. To our knowledge, no human studies have been published on disturbances of tooth mineralization or tooth morphology due to BP administration.

The aim of the present study was to evaluate the effect of BP therapy (i.v. pamidronate) on the development and mineralization of permanent teeth. We hypothesized that BP treatment increases the prevalence of tooth agenesis and causes disturbances in tooth formation/morphology and mineralization when administration is begun in early childhood.

## Methods

### Subjects

The study cohort had all received care at the Astrid Lindgren children’s hospital at Karolinska University Hospital, Stockholm (Sweden’s national multidisciplinary pediatric OI team). In all, 274 children and adolescents with OI were clinically and radiographically examined at several occasions between September 1991 and December 2019 by one of our specialists in pediatric dentistry in the OI team. Forty-five children were excluded due to behavior management problems or low age, of which 3 were diagnosed as OI type V. Ten children did not show up for a radiographic examination. Thus, 219 individuals, 129 boys and 90 girls, were included in the present study and divided into the following groups: group 1, began BP therapy before age 2 years (*n* = 22); group 2, began BP therapy between 2 and 6 years of age (*n* = 20); group 3, began BP therapy between 6 and 10 years of age (*n* = 13); and a control group comprising patients with OI who had never received BP treatment (*n* = 164).

The regional ethics committee in Stockholm approved the study protocol (Daybook no. 157/99 and 2014/254-31/4).

### Clinical Registrations

Type of OI, presence of DGI, tooth agenesis (permanent third molars excluded), disturbances in tooth formation and morphology, anomalies of tooth size and form, and mineralization defects were recorded. Intraoral color photographs were taken at each follow-up.

### Radiographic Analyses

Film format (*n* = 60) or digital panoramic radiographs (*n* = 141) from the dental records of all patients in the study cohort were analyzed regarding DGI, tooth agenesis, and formation of permanent tooth germs. The radiographs were anonymized, and findings were coded using standardized terms. To assess the film format radiographs, we used a Mattsson’s binocular and a light table. Two observers (B.M. and G.T.) evaluated all radiographs. No panoramic radiographs were taken of the 18 patients with all permanent teeth erupted and in occlusion. Two observers, BM and GT, evaluated all radiographs from the four groups separately. BM and GT made a second determination independently. When the scores differed, one of the two dentists (BM or GT) re-evaluated the radiographs.

### Bisphosphonate Therapy

The data extracted from the medical records on BP therapy included doses and duration. The infusions were initially given monthly in doses of 10–30 (40) mg⁄m^2^ pamidronate during 5–8 h preceded by hydration with 25 mg⁄ml of buffered glucose (total dose of 500 ml⁄m^2^ for 2–4 h). For the first 3 months, a dose of 10 mg⁄m^2^ was given; over the next 3 months 20 mg⁄m^2^; and in following months, 30 mg⁄m^2^ for further treatment. Patients with poor treatment response received a dose of 40 mg/m2. Treatment time was later shortened to 4 h without pre-treatment hydration. After Dual-energy X-ray absorptiometry (DXA) confirmed normalization of bone mineral density, treatment intervals were individualized, increasing to once every 2–6 months; doses were decreased depending on bone density values and pain.

### Dental Aberrations

Dentinogenesis imperfecta (DGI) was evaluated from clinical evaluation and from radiographs from both the primary and secondary dentition.

Tooth agenesis was recorded as hypodontia (< six congenitally missing teeth) or oligodontia (≥ six congenitally missing teeth). All participants were followed up until tooth agenesis could be determined.

**Fig. 1:**
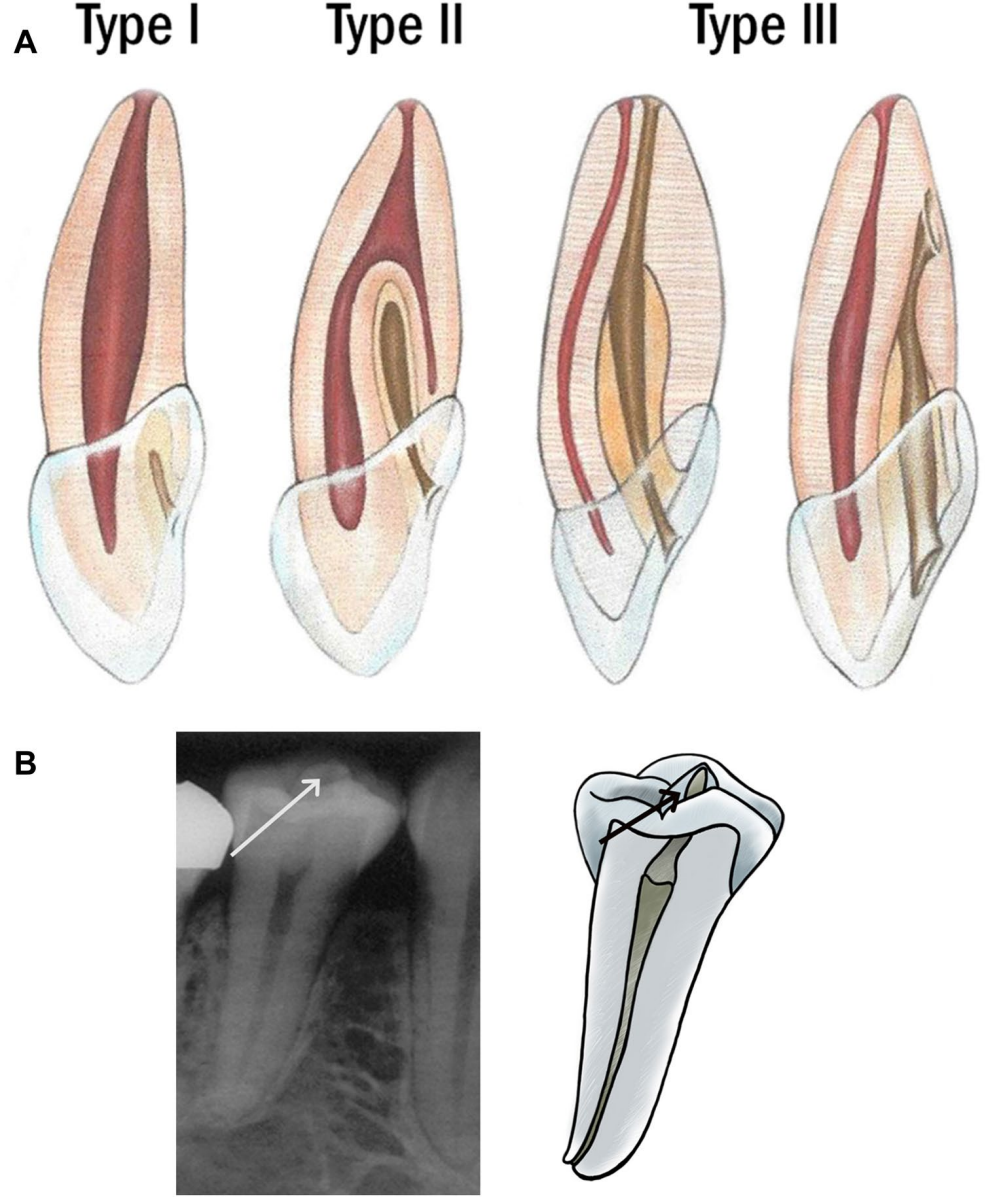
**a** Classification of *dens invaginatus* (Oehlers [17]). A schematic drawing showing the types of invagination according to Oehlers [17]. Type I: an invagination into the crown only. Type II: an invagination into the root that ends in a blind sac. Type III: an invagination that penetrates the root and bursts apically or laterally into a foramen (From Ahmed and Dummer 2017, reproduced with permission from Wiley). **b** *Dens evaginatus*. A radiograph and a schematic drawing of a second lower premolar showing an extra cusp (arrow) with the pulp extending into it

*Dens invaginatus* (DI) was determined according to Oehlers [17]. Only types II and III were registered in upper laterals (Fig. 1a). *Dens evaginatus* (DE) was defined as a tubercle on the occlusal surface consisting of an outer layer of enamel, a core of dentin, and an extension of pulp tissue at least halfway from the cemento-enamel junction to the occlusal surface [18] (Fig. 1b).

Anomalies of tooth size and form (such as microdontia and supernumerary occlusal cusps) were registered clinically and/or radiographically and classified according to the World Health Organization classification system [19]. Enamel disturbances were recorded as hypoplastic or hypomineralized.

The final agreement was based on integration of clinical photos, radiographs, and examination records from several occasions.

### Statistical Analyses

The chi-square test was used for between-group comparisons of the prevalence of tooth agenesis, and for comparisons between the different types of OI. The level of significance was set at 5% (*p*-value < 0.05). All data were analyzed using Statistica v. 13 (StatSoft; Scandinavia AB, Uppsala, Sweden). We randomly selected 20 cases from group 1, 20 from groups 2 and 3, respectively, and 20 from the controls for double determination of DGI, tooth agenesis, dens invagination, dens evaginatus, other abnormalities and enamel defects for intra- and interrater reliability. There was a complete agreement in all positions in the groups 2–4, and in the position DGI and tooth agenesis in group 1. Kappa values were used for inter- and intra-observer agreement between the two observers BM and GT: for DGI and tooth agenesis, an almost perfect agreement (*ĸ* = 0.94), for DI (*ĸ* = 0.84), for enamel defects (*ĸ* = 0.88), and a substantial agreement for DE (*ĸ* = 0.62, and 0.71) and for anomalies in size and form (*ĸ* = 0.69, and 0.63).

## Results

The mean age at imaging in study groups 1, 2, and 3 was 10.1, 8.8, and 10.9, respectively, and in the control group, 10.7 years. Mean ages of BP treatment onset for study groups 1, 2, and 3 were 0.6, 4.1, and 7.8 years, while mean treatment duration was 9.4, 4.6, and 3.1 years, respectively. Table 1 presents the group characteristics.

**Table 1:** Characteristics of the children and adolescents with osteogenesis imperfecta (OI) who *(i)* had received bisphosphonate (BP) treatment (*n* = 55 total among the three groups) and *(ii)* had not received BP treatment (*n* = 164; controls)

	OI type I	OI type III	OI type IV	Total
Group 1: BP therapy onset before 2 years of age				
Number of subjects	3	13	6	22
Gender (M/F)	3/0	3/10	3/3	9/13
DGI (yes/no)	0/3	9/4	3/3	12/10
Age at imaging, years	12.3 (10.6–14.7)	10.3 (5.7–13.8)	9.1 (7.7–12.9)	10.1 (5.7–14.7)
Age at treatment onset, years	0.3 (0.2–0.3)	0.5 (0.2–1.8)	0.7 (0.2.–1.1)	0.6 (0.2–1.8)
Treatment duration at imaging in years	10.1 (7.1–13.0)	9.7 (5.3–12.8)	8.5 (6.8–10.9)	9.4 (5.3–13.0)
Group 2: BP therapy onset between 2 and 6 years of age				
Number of subjects	11	5	4	20
Gender (M/F)	6/5	3/2	1/3	10/10
DGI (yes/no)	1/10	1/4	1/3	3/17
Age at imaging, years	8.2 (6.0–11.9)	9.2 (7.0–11.1)	10.0 (8.9–10.8)	8,8 (6.0–11.9)
Age at treatment onset, years	4.2 (2.3–5.4)	3.7 (2.6–5.9)	4.3 (3.8–4.7)	4.1 (2.3–5.9)
Treatment duration at imaging in years	3.9 (0.7–7.3)	5.3 (1.1–7.8)	5.8 (5.0–6.1)	4.6 (0.7–7.8)
Group 3: BP therapy onset between 6 and 10 years of age				
Number of subjects	8	1	4	13
Gender (M/F)	4/4	0/1	4/0	8/5
DGI (yes/no)	2/6	0/1	1/3	3/10
Age at imaging, years	11.4 (9.0–15.0)	11.4	9.7 (7.6–11.6)	10.9 (7.6–15.0)
Age at treatment onset, years	8.0 (6.9–8.9)	7.1	7.6 (6.2–9.1)	7.8 (6.2–9.1)
Treatment duration at imaging in years	3.4 (1.0–6.2)	4.3	2.1 (1.0–3.3)	3.1 (1.0–6.2)
Controls: no BP therapy				
Number of subjects	131	8	25	164
Gender (M/F)	84/47	2/6	16/9	102/62
DGI (yes/no)	12/119	5/3	9/16	26/138
Age at imaging, years	10.2 (4.2–20.0)	16.9 (8.0–23.7)	11.0 (6.3–19.9)	10.7 (4.2–23.7)

*OI type I* mild OI with blue sclerae, *OI type III* severe deforming, *OI type IV* intermediate severity, and white sclerae after infancy, *DGI* dentinogenesis imperfecta; Age at imaging, treatment onset and treatment duration are given in mean values and range

### Dentinogenesis Imperfecta (DGI)

Dentinogenesis imperfecta (DGI) was found in 20% (*n* = 44) of the 219 patients. It was found in 5 of 8 subjects (63%) with OI type III in group 4 (the controls) and in 9 of 13 subjects (69%) in the study group 1. The prevalence of DGI in children with OI type IV was 50% (3 of 6) in study group 1 and 36% (9 of 25) in the controls. There was no significant difference between groups.

### Tooth Agenesis

Tooth agenesis was found in 14% (*n* = 31) of the 219 participants. The prevalence of tooth agenesis was significantly higher in children who had begun treatment with BP before the age of 2 years (group 1; 59%, *n* = 13), compared to children who had begun BP therapy between ages 2 and 6 years (group 2; 10%, *n* = 2; *p* = 0.009) or after age 6 years (group 3; 8%, *n* = 1; *p* = 0.003), and compared with the controls (9%, *n* = 15; *p* < 0.001; Table 2).

**Table 2:** Tooth disturbances in the children and adolescents with osteogenesis imperfecta (OI) who had received bisphosphonate treatment (*n* = 55 total among the three groups)

Patient	OI type	Age at imaging (years)	Age at last clinical evaluation (years)	DGI^a^	Congenitally missing teeth (tooth number)	DI (tooth number)	DE (tooth number)	Anomalies in tooth size and form (tooth number)	Enamel defects^b^ (tooth number)
Group 1: BP therapy onset before 2 years of age									
1	1	14.7	18	0	15	35	14,24	25,45,	
2	1	13.3	13.3	0	0			15,14,24,25,45,44,34,35	
3	1	10.6	10.6	0	0			35,45,25	
4	3	13.3	11.8	0	14,15,25,35,44,45			34	
5	3	12.3	16.0	1	14,34	15	24,25,44,35	45	
6	3	9.5	7.1	1	0	45		35	
7	3	9.7	9.7	1	15,14,24,25,35,45		34,44		
8	3	7.3	7.5	1	15,24,25,35,45		34		
9	3	12.3	12.3	0	14,15,24,25,35,34				45
10	3	11.5	11.5	1	15,14,24,25,45,35				34,44
11	3	10.0	15.00	1	15,24,25,44		35	14,34,45	
12	3	10.5	10.5	1	14,15,25,34			35	45
13	3	13.8	13.8	0	0		15	35,45,14,24,44,	45,34
14	3	9.0	14.0	1	15,14,24,25,35,34,44,45				
15	3	5.7	5.7	0	0	Evaluation not possible			
16	3	6.4	6.4	1	0	Evaluation not possible			
17	4	11.2	11.2	1	15,25				
18	4	7.9	11.0	0	15,14,24,25,35,34,44,45				
19	4	9.1	16.5	1	0	45,35			
20	4	7.7	7.7	0	15,14,13,24,25			45,35	
21	4	10.0	10.0	1	0			44	35,45
22	4	8.7	12.9	0	0			45	
Group 2: BP therapy onset between 2 and 6 years of age									
1	1	6.6	–	1	0				
2	1	6.0	8.9	0	0				
3	1	11.7	14.6	0	0				
4	1	10	–	0	0				
5	1	11.9	18.1	0	0				
6	1	9.9	12.9	0	0				
7	1	7.3	17.8	0	0				
8	1	6.1	–	0	0				
9	1	8.2	17.5	0	0				
10	1	6.2	–	0	0				
11	1	6.1	8.0	0	0				
12	3	9.2	17.9	0	0				
13	3	8.1	–	1	25				
14	3	7.0	–	0	0				
15	3	11	18.3	0	42,32				
16	3	9.9	9.9	0	0				
17	4	9.7	–	0	0				
18	4	8.9	8.9	1	0				
19	4	10.7	11.7	0	0				
20	4	10.8	7.3	0	0				
Group 3: BP therapy onset between 6 and 10 years of age									
1	1	11	11.5	0	0				
2	1	10.9	12.8	0	0				
3	1	10.3	8.4	0	0				
4	1	15	8.7	1	0				
5	1	9	10.0	1	0				
6	1	11.6	–	0	0				
7	1	9.2	14.7	0	0				
8	1	14.5	12.0	0	0				
9	3	11.4	11.4	0	0				
10	4	8.7	13.1	0	0				
11	4	7.6	7.6	0	15, 45				
12	4	11.6	15.5	0	0				
13	4	10.8	16.7	1	0				

^a^0 = no, 1 = yes

^b^hypoplasia/hypomineralization

*OI type I* mild OI with blue sclerae, *OI type III* severe deforming, *OI type IV* intermediate severity, and white sclerae after infancy, *DGI* dentinogenesis imperfect, *DI* Dens invaginatus, *DE* Dens evaginatus

Tooth numbers: 15 = right, second upper premolar; 14 = right, first upper premolar; 24 = left, first upper premolar; 25 = left, second upper premolar; 35 = left, second lower premolar; 34 = left, first lower premolar; 32 = left, second lower, incisor; 42 = right, second lower, incisor; 44 = right, first lower premolar; 45 = right, second lower premolar

**Fig. 2:**
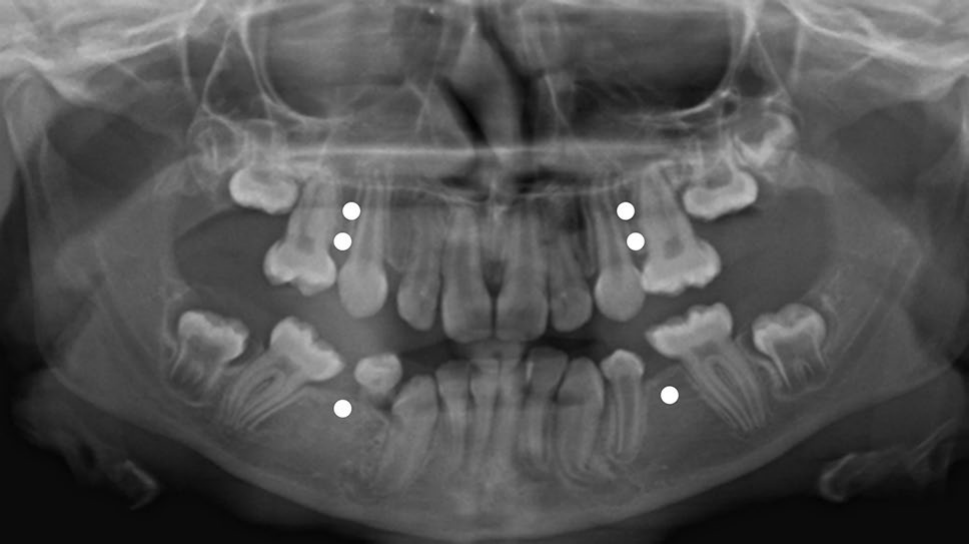
An 11.5-year-old girl with osteogenesis imperfecta (OI) type III and dentinogenesis imperfecta (DGI; patient no. 10). Six premolars are missing congenitally. Circles mark the sites of missing germs

There were no significant differences in tooth agenesis between the controls and those with BP onset between 2 and 6 years or after 6 years of age. Oligodontia was found in 8 of 219 subjects (4%) of which 6 were found in study group 1 (27%) and 2 in the control group (1%) (Fig. 2). In the control group, besides the two patients with oligodontia, five patients were missing only one premolar, one was missing the upper laterals, four were missing two premolars, and three were missing four premolars. The prevalence of tooth agenesis was significantly higher in children with OI type III who had begun treatment before the age of 2 years (group 1; 69%, *n* = 9) compared to those treated after 2 years of age or not treated (group 2, 3, 4; 27%, *n* = 3; *p* < 0.013). The prevalence in children with OI type IV who had begun treatment before the age of 2 years (group 1; 50%, *n* = 3) was also significantly higher compared to those treated after 2 years of age or not treated (group 2, 3, 4; 9%, *n* = 3; *p* < 0.011). The children with OI type I in group 1 (*n* = 3) were too few for statistical analysis.

### Tooth Formation/Morphology

**Fig. 3:**
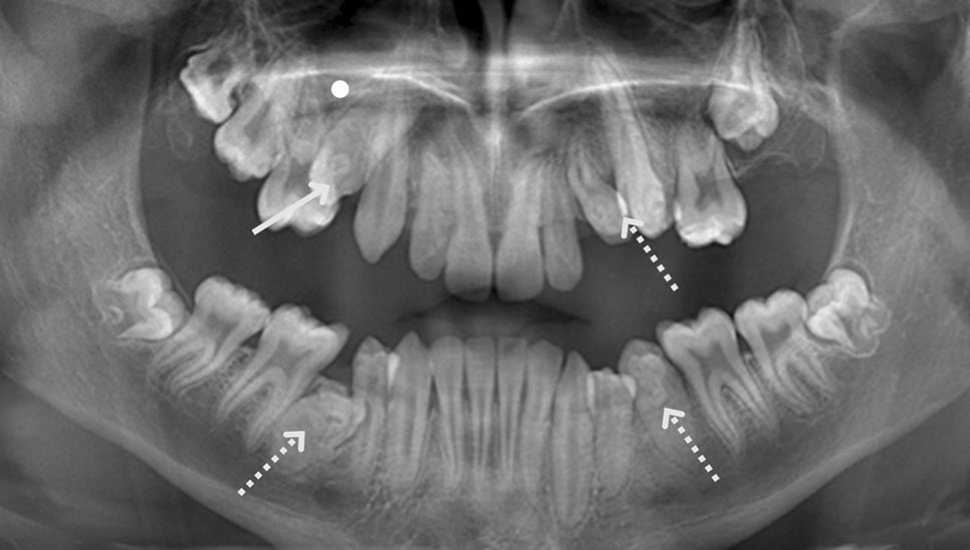
A 14.7-year-old young adult with severe osteogenesis imperfecta (OI) type I without dentinogenesis imperfecta (DGI; patient no. 1). The panoramic radiograph shows one congenitally missing tooth in the right upper jaw (the site of the missing germ marked with a circle) and invagination of an upper right premolar (continuous arrow). Premolars with *dens evaginatus* or other malformations are marked with broken arrows. One premolar in the upper left jaw had been extracted due to crowding

Malformed premolars with affected tooth formation and morphology such as DI and DE were found in 7 of the 22 (32%) children treated with BPs before the age of 2 years (Fig. 3). All cases where DI was diagnosed represented Oehlers type II, that is, an invagination into the root that ends in a blind sac. Only one patient in the control group was diagnosed with a defect in tooth formation (DI). In cases of DE where clinical diagnosis was possible, all premolars had a tubercle on the occlusal surface and an extension of pulp tissue at least halfway from the cemento-enamel junction to the occlusal surface (Table 2).

### Anomalies of Tooth Size and Form

Premolars with a disturbance in tooth size and form, such as microdontia and supernumerary occlusal cusps, were found in 12 of 22 (55%) children who had received BP treatment before the age of 2 years. No anomalies of tooth size or form were found in the other groups (Table 2).

### Mineralization Disturbances

**Fig. 4:**
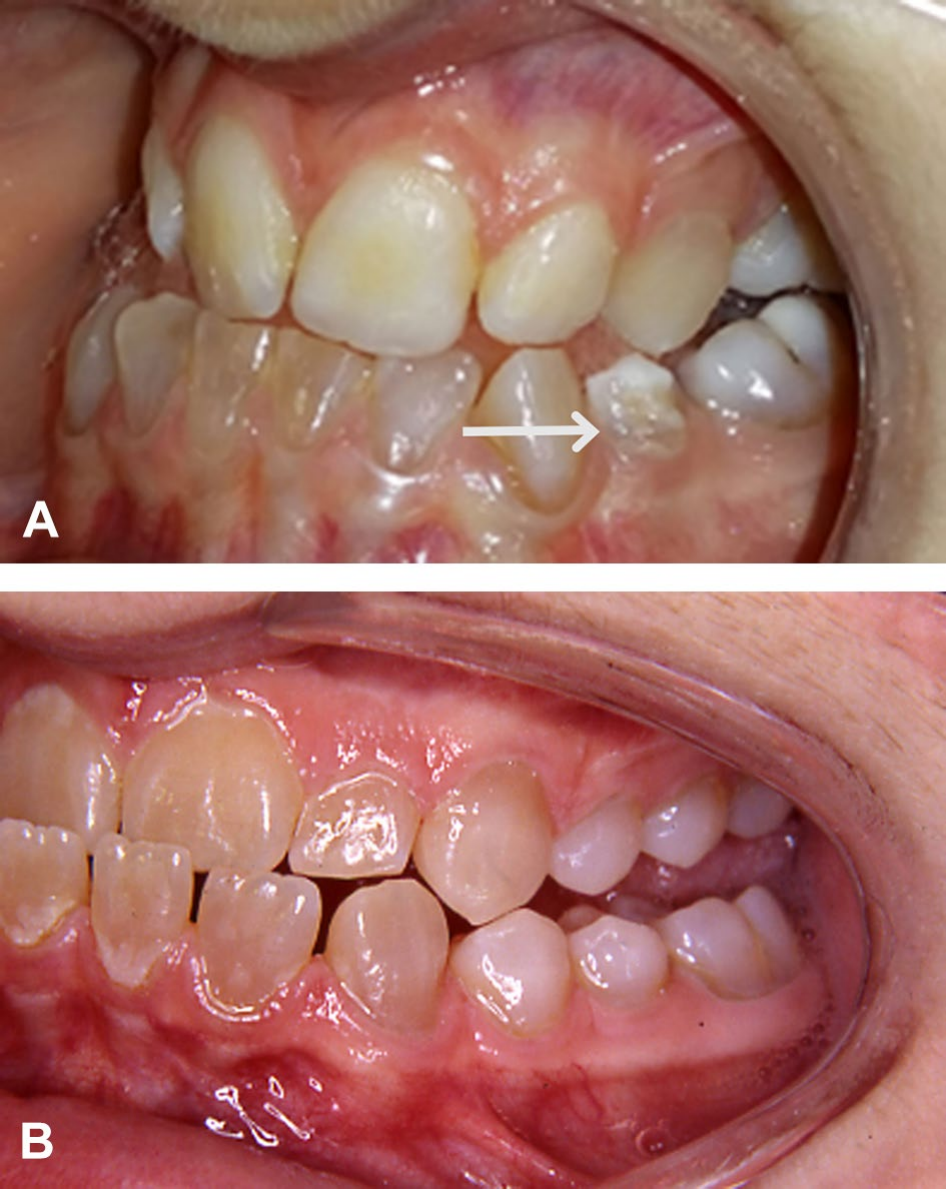
**a** An 11.5-year-old girl with osteogenesis imperfecta (OI) type III and dentinogenesis imperfecta (DGI; patient no. 10). Enamel hypoplasia is visible on the lower left premolar. All premolars in the upper jaw, along with the second premolars in the lower jaw, are congenitally missing. BP treatment began when the girl was 0.29 years old. **b** A 19-year-old man with OI type IV and DGI (a control) who has received BP therapy from the age of 17 years. No enamel disturbances are visible

Teeth with enamel hypoplasia and enamel hypomineralization were clinically diagnosed and found in 5 of 22 individuals with erupted premolars (23%) treated with BPs before the age of 2 years. No such disturbances were found in any of the other groups (Fig. 4; Table 2).

## Discussion

Our main findings are that BP administration can affect tooth development when treatment starts before 2 years of age. Prevalences of tooth agenesis, malformed teeth, and tooth mineralization disturbances were significantly higher in children who had received BP treatment from infancy compared to children who had begun BP treatment first after the age of 2 years and those who had not received BP therapy. Tooth agenesis and DGI is most frequently seen in individuals with OI type III. DGI is not influenced by BP treatment. As OI type III is the most severe type of OI, BP treatment usually starts early in these patients. We found the prevalence of tooth agenesis significantly higher in children with OI type III who had begun treatment before the age of 2 years compared to those treated after 2 years of age or not treated.

In most previous studies, the average age of children with OI at onset of BP therapy was around 4 years. However, there are reports of children who began treatment as early as 2 weeks of age [15]. The most widely used BPs in pediatric patients are pamidronate and zoledronate. They have different in vitro potencies with pamidronate having the lowest relative potency (100) and zoledronic acid the highest relative potency (10,000). At the present time, possible negative effects of BPs on tooth development are being debated. Based on reported in vitro data relevant for dentistry, clinical use of etidronate with the lowest relative potency should cease due to its impact on tooth development, whereas use of alendronate with the relative potency between 1000–2000 seems to be safe [20]. To our knowledge, the present study is the first to focus on the impact of BP therapy on tooth development in humans.

Third molar agenesis is the most common form of agenesis and being the most variable tooth in the dentition with its formation time and morphology. The permanent third molars are excluded in all Nordic prevalence studies and in most European. Nordic studies have reported tooth agenesis prevalences, excluding third molars, of between 6 and 8% [21] and oligodontia prevalences of about 0.1–0.2% [22]. Mutations in several genes have been associated with familial tooth agenesis, among others *MSX1*, *PAX9*, *AXIN2*, *EDA, EDARADD,* and *EDAR* [23, 24]. Oligodontia has been reported in individuals with OI [4, 25, 26]. In a Finnish study on patients with OI before BP therapy had been initiated, Lukinmaa et al. [25] reported tooth agenesis in 18.4% of the cohort and oligodontia in 2%. The Malmgren et al. [4] study found hypodontia in 11% and oligodontia in 6% of their patients with OI. In that study, treatment with BPs was not considered; instead, the very high prevalence of hypodontia and oligodontia prompted the group to investigate whether mutations in genes other than those earlier associated with OI (*COL1A1*, *COL1A2*, and *CREB3L1*) were responsible for the disturbances [27]. Except for the known variants, we were unable to identify any other mutual variant related to collagen type I that could explain the phenotype of OI associated with hypodontia/oligodontia. In the present study, we found that 31 (14%) of all patients with OI had tooth agenesis, and 13 of the 31 had been treated with BPs before the age of 2 years. Furthermore, eight (4%) of all patients with OI exhibited oligodontia, and six of them had begun treatment with BPs before the age of 2 years. Oligodontia is a serious condition and negatively affects quality of life [28, 29]. Since onset of BP treatment in early childhood may cause this condition, their use should be carefully considered before BP therapy is begun in newborns and infants.

*Dens invaginatus* (DI) and *Dens evaginatus* (DE) are developmental aberrations often causing pulp necrosis if not prophylactically treated. DI forms by invagination of the enamel organ into the dental papilla prior to calcification of the dental tissues [30]. We found DI in 20% of group 1, children treated before the age of 2 years. This is high compared to a study of 3020 Swedish schoolchildren where DI was found radiographically in the upper incisors in 2.7% of the group; in 43% of these cases, DI was bilateral [31] and predominantly type I according to Oehlers [17]. DI rarely occurs in premolars. Ridell et al. [32] collected all dental records from the Department of paediatric dentistry at the Eastman Dental Institute in Stockholm with a diagnosis of DI between 1969 and 1997, (*n* = 131 teeth). Only one invagination was observed in a premolar (0.8%) compared with 20% in group 1 in the present study. Since DI in permanent premolars is rare, only a few case reports have been published [33].

DE is a rare developmental anomaly occurring on the crown of the tooth as an extra bump or cusp, often with a fine pulpal extension. Premolars are more frequently affected than other teeth [18]. Lin et al. [34] studied two groups of age- and sex-matched individuals, 147 Taiwanese and 147 Spanish students. DE was found in 4.1% in the Taiwanese group but none in the Caucasian group, which the authors thought could possibly suggest that DE may be common in Asian populations. We found DE in 25% in group 1 and none in the other groups. This high number of DE in Caucasians has not been previously reported.

Interestingly, besides DI and DE, striking morphological defects were observed in the second premolars of patients with OI who had received BP therapy before the age of 2 years. The cusps of the second premolars were abnormal in size and patterning, with mineralization defects of the enamel a common occurrence. To our knowledge, a similar phenotype has not been reported previously. The serial formation of teeth during development, where the second premolars are the last teeth to form and their crown development is incomplete at age 2 years, would explain the exceptional vulnerability of the second premolars [35]. In our study, BP treatment of all children in group 1 began before 2 years of age, which is earlier than in the study cohorts of most published studies. While the morphogenesis of most organs occurs during the embryonic stage, tooth formation continues postnatally, which would explain why BP therapy affects tooth morphogenesis but has no detected effect the development of other organs.

The observed associations of *dens invaginatus* (DI) and *dens evaginatus* (DE) with dentinogenesis imperfecta (DGI) are likely explained by the disturbed structure of forming dentin in DGI. This abnormal dentin may have affected the next dental epithelium and its folding which determines the tooth cusp patterns. The resulting abnormal epithelial invaginations and evaginations would finally develop to DI and DE, respectively.

The presence of developmental defects of the enamel was determined clinically, and the defects were classified as hypoplastic or hypomineralized. The buccal surface of the tooth crown was the site that was most affected. Animal studies have reported indications of disturbances in tooth eruption and mineralization due to BP administration [36–39], but to our knowledge our study is the first to report the effect of pamidronate on enamel formation in humans.

Tooth eruption requires osteoclastic bone resorption [40]. Hiraga et al. [38] suggested that apoptosis induction of the osteoclasts by nitrogen-containing BPs (NBPs) impairs tooth eruption in zoledronate-treated rats. The study also found a possible relationship between zoledronate administration and induction of several types of dental abnormalities; it demonstrated that un-resorbed bone particles occasionally came into contact with or even compressed the cells of the enamel organ in zoledronate-treated rats. This pressure was suggested to affect ameloblasts and enamel matrix formation in the incisors, leading to enamel defects.

Developmental disturbances in tooth morphology and mineralization due to administration of etidronate and alendronate have also been reported in animal studies [36, 38, 39, 41, 42]. In a scanning electron microscope study [36] of 72 alendronate-treated newborn rats, morphological alterations such as depressions were detected along the entire enamel surface and at the cervical portion. In rats, etidronate treatment [39] was shown to cause disturbances in the ameloblasts and developing enamel, resulting in enamel hypoplasias.

Tooth development comprises highly regulated complex events characterized by cell–cell and cell–extracellular matrix (ECM) interactions [43]. These interactions regulate tooth shape and size as well as the differentiation of odontoblasts and ameloblasts, which form dentin and enamel, respectively [20]. Tooth development and eruption are accompanied by remodeling of the adjacent bone, which is deposited by osteoblasts and resorbed by osteoclasts, and serum calcium and phosphate are attracted to form hydroxyapatite in the developing tooth structure [40]. Between 30 and 70% of the absorbed i.v. administered dose of BP is deposited in the skeleton at sites of active bone remodeling [44]. For these reasons, BPs may affect tooth development and eruption.

In conclusion, BP therapy has improved the quality of life considerably [45], but the treatment does not seem to have a positive effect on dental aberrations, which are common in individuals with OI. The results of the present study indicate that pamidronate treatment started before the age of 2 years increases the risk of disturbances in tooth development. We have followed up 22 children, treated with BP before 2 years of age, until all permanent teeth could be evaluated. Although the group is small, the results indicate that serious disturbances in tooth formation occur. Collaborations with other centers could further support the findings. Individuals with OI are in need of early multidisciplinary treatment planning; therefore, disturbances in dental development should be diagnosed as soon as possible.

### Limitations

BP treatment usually starts early in patients with the most severe type of OI, OI type III, and 59% of the subjects in group 1 (BP starts before 2 years of age) were diagnosed as OI type III. Not surprisingly, there are a large number of patients with OI type I in control group 1. This type of OI is the mildest and most common form. Three cases with OI type I with repeated fractures and acquired vertebral fractures at treatment start were included in group 1 but the number of patients was too small in this group for analysis. Although the results must be viewed with caution, the seriously affected premolars seen in one of the patients with OI type I (Fig. 3) is striking. Treatment with BP started in 1991 first in adolescents and young adults, which explains the low number of OI type III in groups 2 and 3 (*n* = 6) and in the controls (*n* = 8). However, none of these 14 patients showed any other tooth abnormalities but a few congenitally missing permanent teeth in three cases.

In two cases in group 1 (BP before the age of 2 years), assessment of dental aberrations was difficult to provide due to overlapping contours. These were only included for tooth agenesis assessment. Statistical analysis was difficult to interpret when the groups were categorized into the different OI types. Therefore groups 2, 3, and 4 were pooled.
